# Identifying stroke therapeutics from preclinical models: A protocol for a novel application of network meta-analysis

**DOI:** 10.12688/f1000research.15869.1

**Published:** 2019-01-03

**Authors:** Manoj M. Lalu, Dean A. Fergusson, Wei Cheng, Marc T. Avey, Dale Corbett, Dar Dowlatshahi, Malcolm R. Macleod, Emily S. Sena, David Moher, Risa Shorr, Sarah K. McCann, Laura J. Gray, Michael D. Hill, Annette O'Connor, Kristina Thayer, Fatima Haggar, Aditi Dobriyal, Hee Sahng Chung, Nicky J. Welton, Brian Hutton

**Affiliations:** 1 Department of Anesthesiology and Pain Medicine, The Ottawa Hospital, Ottawa, Canada; 2 Clinical Epidemiology Program, Blueprint Translational Research Group, The Ottawa Hospital Research Institute, Ottawa, Canada; 3 Department of Cellular and Molecular Medicine, University of Ottawa, Ottawa, Canada; 4 Regenerative Epidemiology Program, The Ottawa Hospital Research Institute, Ottawa, Canada; 5 School of Epidemiology and Public Health, University of Ottawa, Ottawa, Canada; 6 Knowledge Synthesis Group, Clinical Epidemiology Program, The Ottawa Hospital Research Institute, Ottawa, Canada; 7 Heart & Stroke Foundation Canadian Partnership for Stroke Recovery, University of Ottawa, Ottawa, Canada; 8 Department of Medicine. Division of Neurology, The Ottawa Hospital, Ottawa, Canada; 9 Neuroscience Program, The Ottawa Hospital Research Institute, Ottawa, Canada; 10 Centre for Clinical Brain Sciences, University of Edinburgh, Edinburgh, UK; 11 Learning Services, The Ottawa Hospital, Ottawa, Canada; 12 Department of Health Sciences, University of Leicester, Leicester, UK; 13 Cumming School of Medicine, Clinical Neurosciences and Hotchkiss Brain Institute, University of Calgary, Calgary, Canada; 14 College of Veterinary Medicine, Iowa State University, Ames, Iowa, USA; 15 National Institutes of Environmental Health Sciences, Durham, North Carolina, USA; 16 Faculty of Medicine, University of Ottawa, Ottawa, Canada; 17 Department of Population Health Sciences, Bristol Medical School, University of Bristol, Bristol, UK

**Keywords:** stroke, preclinical, systematic review, network metaanalysis, network meta-analysis

## Abstract

**Introduction:** Globally, stroke is the second leading cause of death. Despite the burden of illness and death, few acute interventions are available to patients with ischemic stroke. Over 1,000 potential neuroprotective therapeutics have been evaluated in preclinical models. It is important to use robust evidence synthesis methods to appropriately assess which therapies should be translated to the clinical setting for evaluation in human studies. This protocol details planned methods to conduct a systematic review to identify and appraise eligible studies and to use a network meta-analysis to synthesize available evidence to answer the following questions: in preclinical *in vivo* models of focal ischemic stroke, what are the relative benefits of competing therapies tested in combination with the gold standard treatment alteplase in (i) reducing cerebral infarction size, and (ii) improving neurobehavioural outcomes?

**Methods:** We will search Ovid Medline and Embase for articles on the effects of combination therapies with alteplase. Controlled comparison studies of preclinical *in vivo *models of experimentally induced focal ischemia testing the efficacy of therapies with alteplase versus alteplase alone will be identified. Outcomes to be extracted include infarct size (primary outcome) and neurobehavioural measures. Risk of bias and construct validity will be assessed using tools appropriate for preclinical studies. Here we describe steps undertaken to perform preclinical network meta-analysis to synthesise all evidence for each outcome and obtain a comprehensive ranking of all treatments. This will be a novel use of this evidence synthesis approach in stroke medicine to assess pre-clinical therapeutics. Combining all evidence to simultaneously compare mutliple therapuetics tested preclinically may provide a rationale for the clinical translation of therapeutics for patients with ischemic stroke.

**Dissemination**: Review findings will be submitted to a peer-reviewed journal and presented at relevant scientific meetings to promote knowledge transfer.

**Registration:** PROSPERO number to be submitted following peer review.

## List of abbreviations

NMA, network meta-analysis; DIC, deviance information criteria; NMD, normalized mean difference

## Introduction

Globally, an estimated 15 million people suffer a stroke; stroke is the second leading cause of death, with six million people dying and an additional five million becoming permanently disabled each year ^1, 2^. The costs of stroke are high due to a combination of immediate high costs from acute care and long-term costs from resulting disability. Worldwide cost estimates range from $266 billion to $1.038 trillion per year ^3^. Despite the enormous human and economic burden, only four acute interventions ** are currently used clinically: patient care in a dedicated stroke unit ^3^, reperfusion (by pharmacological thrombolysis or endovascular mechanical thrombectomy ^4^), oral aspirin, and surgical decompression.

In the search for novel therapies for acute stroke, more than 1,000 potential neuroprotective therapeutics (e.g. anticoagulants, calcium channel blockers, free radical scavengers, GABA mimetics, etc.) have been evaluated in preclinical models ^5^. Of these, only reperfusion with tissue plasminogen activators ^6^, such as alteplase ^3^, has had a preclinical basis. Despite its efficacy, alteplase has inherent limitations such as the risk of hemorrhagic transformation, which warrants exploration of novel adjunctive therapies that can maximize therapeutic benefit. Combination therapies with alteplase might limit reperfusion injury and cell death that can sometimes occur with this drug. However, given the multitude of therapies tested preclinically (and multiple mechanisms of action) it is difficult to assess which therapies should proceed to clinical testing.

Preclinical systematic reviews have served as a robust form of knowledge synthesis to evaluate transparently experimental therapies for more than a decade ^7– 9^. Previous preclinical systematic reviews have compared treatments in isolation using pair-wise meta-analyses, which limits the ability to simultaneously evaluate comparative effectiveness in the presence of many treatments of interest. Use of network meta-analysis (NMA) in comparative effectiveness research to study the relative benefits and harms of multiple interventions in humans ^9, 10^ has risen dramatically during the past decade ^11^. Such analyses allow the comparison of many interventions based on all ‘direct’ and ‘indirect’ information. In addition, this approach has the potential to establish a more rigorous framework for decisions to embark on clinical trials while reducing risks to human trial participants and the enormous costs of preclinical translation ^3, 7, 12^. Comparison of preclinical stroke therapeutics represents an excellent case study for such work. Given the novelty of this approach, this systematic review will also serve as a case study to empirically explore the methodological nuances of applying NMA in a preclinical setting.

### Protocol

This protocol will be registered in the international prospective register of systematic reviews (PROSPERO, CRD) following peer review. Our review protocol is reported in accordance with the Preferred Reporting Items in Systematic reviews and Meta-Analysis-Protocol guidelines (a complete checklist is available as Supplementary File 1) ^13^. Post-protocol adjustments will be included in the final report.

### Objectives

***Primary objective.*** We will perform a systematic review and NMAs to address the following question: amongst *in vivo* models of focal ischemic stroke, what are the relative benefits of competing therapies tested in combination with the gold standard treatment alteplase ^14^ in (i) reducing cerebral infarction size, and (ii) improving neurobehavioural outcomes?

***Secondary Objective.*** We will also (i) assess the risk of bias of the included studies, and (ii) explore what novel considerations for statistical adjustments are necessary for NMA of preclinical studies (e.g. method of ischemic induction, timing of treatment, species, sex, and comorbidities). We will also evaluate the challenges of applying NMA to preclinical studies (e.g. consistency, heterogeneity, availability of key study covariates).

## Methods

### Search and study identification

An information specialist (RS) will construct a search strategy based on a previous review of comparative stroke therapies, and limit them to include studies which compared therapies to alteplase (representative search strategy is provided in Supplementary File 2) ^15^. Search strategies will be peer reviewed by a second information specialist using the peer review of electronic search strategy method ^16^. Searches of Ovid MEDLINE and Embase will be carried out for articles on the effects of combination therapies with alteplase ( Supplementary File 2 contains the search strategy). Of note, no language or date restrictions will be used. We will also search the CAMARADES database which contains data extracted from existing preclinical systematic reviews on stroke ^15, 17– 28^. In addition to this rigorous search, we will assess bibliographies of any new studies and reviews identified. Articles in foreign languages will be translated.

### Study eligibility criteria

Eligibility criteria to identify relevant studies for the current review were established in considering the Population-Intervention-Comparators-Outcomes-Study design (PICOS) framework ^29^.

***Population.*** Preclinical *in vivo* models of experimentally induced focal ischemia will be sought. All species/strains of animals will be eligible. Both female and male animals will be included. Neonatal animals will be excluded; however, all other ages will be considered. Studies in which focal ischemic stroke was established by transient occlusion of the middle cerebral artery or anterior cerebral artery via any method (chemical, embolic, mechanical, thermal) will be eligible. Animal models of haemorrhagic stroke, global or hemispheric brain ischemia, models of permanent occlusion without reperfusion (e.g. photothrombosis, cauterization), or delayed reperfusion such that it is considered permanent will be excluded ^30^. Human studies and tissue culture studies will be excluded.

***Intervention and comparator.*** Studies where the treatment in combination with alteplase (e.g. alteplase + hypothermia) is compared with alteplase alone in animals that have experimentally induced focal ischemia will be eligible. Studies that compare more than one active treatment such as alteplase + hypothermia versus alteplase +FK506 (i.e. head to head comparisons) will also be included. Studies must include alteplase as a ‘foundational’ therapeutic in experimental arms to be eligible. All delivery routes and doses will be considered. To increase potential clinical relevance (i.e. construct validity), only studies that deliver therapies within 6 hours of induction of focal ischemic stroke will be included.

### Outcome measures

- ***Primary outcome***. *Infarct size* is a measure of injury reduction at the infarct site in the brain and can be measured via a variety of quantifiable techniques through non-invasive techniques (e.g. T _2_-weighted magnetic resonance imaging) or post-mortem analysis (e.g. staining of brain sections using hematoxylin and eosin). This is the most widely reported outcome in preclinical stroke studies. Infarct size outcomes will be extracted at the latest time point for each study. Separate time-point specific analyses will be conducted (e.g. an early time point <30 days vs later time points >30 days).
- ****Secondary outcome****. *Neurobehavioural* measures represent a valuable means of assessing functional recovery after treatment. Neurobehavioural assessment are sensitive to detecting the array of impairments, including motor/sensory deficits (e.g. ladder rung walking—foot slip errors) as well as memory/learning deficits (e.g. Morris water maze) ^31, 32^. These outcomes, while labour-intensive, are typically reported with less frequency than infarct volume even though functional outcomes may have the greatest clinical relevance) ^33, 34^. Neurobehavioral outcomes will be extracted at all timepoints and separate time-point specific analyses will be conducted as described above.

### Study design

Controlled comparison studies testing the efficacy of therapies + alteplase versus alteplase alone will be sought.

### Screening and study selection

Two reviewers (A.D. and H.S.C.), will review abstracts (Stage 1 screen) and full text reports (Stage 2 screen) from search results independently and in duplicate against the eligibility criteria below using Distiller SR® software (Evidence Partners, Ottawa, ON) to identify relevant articles. Discrepancies will be resolved through discussion with a senior team member (M.L. and D.C.). Both stages of screening will begin with a calibration exercise to ensure consistent application of eligibility criteria. A PRISMA flow diagram ^35^ will be presented to document the process of study selection.

### Data extraction

Two independent reviewers (A.D. and H.S.C.) will review studies and extract data into standardized, piloted forms implemented in Microsoft Excel (Microsoft Corporation, Seattle, Washington, USA). Discrepancies will be resolved through discussion with a senior team member. We will collect data related to, but not limited to, animal characteristics ( Table 1); stroke model ( Table 1); intervention ( Table 2a, b); and outcomes ( Table 3), as well as study ID (authors, year), and study design characteristics. Measures of central tendency (e.g. mean) and dispersion (e.g. standard deviation) will be extracted as reported. Data in graphical format will be extracted using Engauge Digitizer ^36^. When measures of central tendency and dispersion or sample sizes are missing (or cannot be measured digitally), authors will be contacted; if authors do not respond, the data will be excluded.

**Table 1.** Study characteristics.

Question	Responses
Exclude Study	Yes (provide reason for exclusion) or No
RefID	Text
First Author	Text
Year of Publication	Text
Correspondence (Author, Email)	Text
Funding Support	Not Reported Government Industry Academic Institution Charity Foundation Other Unclear
Country of Corresponding Author	Canada China Japan South Korea United States Other Unclear
Species	Mouse Rat Rabbit Swine Mini-Swine Sheep Dog Monkey Other
Strain	Text
Sex	Male, Female, Both or Unclear
*If Both*	Proportion of Male and Female Stated (Text)
Weight	Text or Not Reported
Age	Text or Not Reported
Type of Model	Intraluminal Suture MCA Embolism Photothrombosis Endothelin 1 Vasoconstriction Tamura MCA Clip Small Vessel Stroke Other Not Reported
Duration of Follow Up from Initiation of Disease State	Text with Units or Unclear

**Table 2.** Study interventions with alteplase.

a. Combination therapy with alteplase	
Questions	Responses
N Initially Reported?	Text
Time to Alteplase Administration Post- Stroke (h)?	Time Provided (Text) Unclear
Frequency of Alteplase Administration?	Single Dose Multiple Dose (Text) Unclear
Dose of Alteplase Delivered	Text
Category of the Comparative Therapy	Nonpharmacological Pharmacological Reperfusion Surgical Cell Based Therapy Other
Comparative Therapy Type	Abciximab, Albumin, Alpha-PBN, Annexin, Anti-CD18, Aortic Occlusion, Argatroban, Atorovastatin, Citicoline, Clopidogrel, CP101,606-27, Dizocilpine, Edaravone, Eliprodil, Enlimonab, EPO, Estrogen, GCSF, Heparin, Hypothermia, Insulin, MC-1, Melagatran, Melatonin, Minocycline, NBQX, Normobaric Oxygen, Pentasaccharide, Pravastatin, PS519, Rosiglitazone, Rosuvastatin, S-0139, Tacrolimus, Tirilizad, TS-011, UK-279, 276, Velcade, XG-102, YM872, Other (Text)
Was the comparative therapy delivered before, with, or after alteplase?	Prior to Administering Alteplase In Conjunction with Alteplase After Delivering Alteplase Unclear
Time to Comparator Administration Post-Stroke (h)?	Time Provided (Text) Unclear
Frequency of Comparative Therapy Administration	Single Multiple (Text) Other (Text) Unclear (Text)
Dose of Comparative Therapy Delivered	Text
Mode of Comparative Therapy Delivery	Intravenous Intra-Arterial Oral Other (Text) Not Reported
b. Alteplase monotherapy	
Question	Response
N Reported	Text
Monotherapy Type	Abciximab, Albumin, Alpha-PBN, Annexin, Anti-CD18, Aortic Occlusion, Argatroban, Atorovastatin, Citicoline, Clopidogrel, CP101,606-27, Dizocilpine, Edaravone, Eliprodil, Enlimonab, EPO, Estrogen, GCSF, Heparin, Hypothermia, Insulin, MC-1, Melagatran, Melatonin, Minocycline, NBQX, Normobaric Oxygen, Pentasaccharide, Pravastatin, PS519, Rosiglitazone, Rosuvastatin, S-0139, Tacrolimus, Tirilizad, TS-011, UK-279, 276, Velcade, XG-102, YM872, alteplase
Time to Monotherapy Administration Post-Stroke?	Time Provided (Text) or Unclear
Dose of Monotherapy Delivered	Text
Frequency of Administration of Monotherapy	Single, Multiple (Text), Other, Unclear
Mode of Monotherapy Delivery	Intravenous, Intraarterial, Oral, Other (Text), Unclear

**Table 3.** Outcomes.

Question	Responses
*If outcome was measured, indicate the latest time point measured in days.*	
Infarct Volume (Primary Outcome of Interest)	Yes, No If yes, indicate latest time point
Neurobehavioral Outcomes Measured (Secondary Outcome of Interest)	
Walking Test	Yes, No If yes, indicate latest time point
Forelimb Asymmetry Tests	Yes, No If yes, indicate latest time point
Skilled Reaching Tests	Yes, No If yes, indicate latest time point
Adhesive Removal Test	Yes, No If yes, indicate latest time point
Measures of General Neurological Status: Neurological Severity Scores (mNSS)	Yes, No If yes, indicate latest time point
Measures of General Neurological Status: Rotarod	Yes, No If yes, indicate latest time point
Other Relevant Neurobehavioral Test	Yes (Text), No If yes, indicate test used and time point

### Assessment of risk of bias and construct validity

Two independent reviewers will assess the risk of bias of each included study (quality of the design, conduct and analysis for the experiment) ^37^. We will assess the risk of bias using a modified version of the Cochrane Risk of Bias Tool for randomized trials ( Table 4). Risk of bias will be summarized ^38^ with descriptive statistics and presented graphically using standard methods and radar charts. The assessment of risk of bias will play an important role in exploring potential limitations of the evidence base and establishing the feasibility of incorporating relevant adjustments in NMA models. The construct validity of included studies (i.e. degree to which experimental model and design reflect the clinical entity of stroke and its treatment) will be assessed using elements from the CAMARADES checklist alongside criteria established by expert consensus ( Table 5).

**Table 4.** Risk of bias.

Question	Responses
Sequence Generation	Low risk = Randomization was mentioned and good method used High risk = Randomized but poor method used High risk = Non-randomized Unclear risk = Randomized but no method described Unclear risk = No mention of randomized or non-randomized
Allocation Concealment	Low risk = Method used to conceal the allocation sequence is described in sufficient detail Unclear risk = Insufficient information to determine if the allocation sequence was concealed High risk = The allocation sequence was not concealed or was concealed in a poor manner
Blinding of Personnel	Low risk = All personnel involved in giving intervention were blinded to the study groups Unclear = Insufficient information to determine if any personnel giving intervention were blinded to the study groups High risk = All personnel giving intervention were described to be unblinded to the study groups
Blinding of Outcome Assessment	Low risk (all) = Outcome assessors were blinded to the study groups for each outcome assessed Low risk (some) = Outcome assessors were blinded to the study groups for at least one outcome assessed. Select the outcomes that were blinded Unclear = Insufficient information to determine if outcome assessors were blinded during assessment High risk = Outcome assessors not blinded to the study groups
Incomplete Outcome Data	Low risk = N values were consistent between methods and results for all outcomes, or inconsistent N values were explained (e.g. only N=3 animals were selected for histological analysis) Unclear = The N value was either not presented in the methods or in the results, and therefore there is insufficient information to permit judgment High risk = N values were not consistent between methods and results for the final outcomes without explanation of attrition or were inconsistent between outcomes
Potential Bias due to the Source of Funding	Low risk = Non-industry source of funding (or no funding) High risk = Any industry source of funding Unclear = Funding source not reported
Potential Bias due to the Sample Size Calculation	Low risk = Sample size calculations were correctly performed and followed High risk = Sample size calculations were incorrectly performed or followed Unclear = Sample size calculations were not reported
Potential Bias due to Reported Conflict of Interest	Low risk = Authors reported no conflict of interest High risk = Authors reported potential conflict of interest Unclear = Potential conflicts of interest not reported

**Table 5.** Construct validity.

Question	Responses
Was an adult animal used? Rats: ≥ 6 weeks Mice: ≥ 8 weeks Rabbit: ≥ 6 months Sheep: ≥ 38 weeks Dog: ≥ 6 months Cats: ≥ 6 months Minipig: ≥6 months Swine: ≥ 6 months Monkey (Macaques): ≥ 4 years	Yes: Age was explicitly reported Yes: Study only mentions “adult” No: Age was explicitly stated but is under the standard “adult age” Unclear: Age was not reported Unclear: Age and weight unreported (but not labelled as a neonate)
Animals Present with Comorbidities Commonly Associated with Ischemic Stroke?	Yes (Text) No Unclear
Avoidance of Anesthetics with Neuroprotective Effects (i.e. Ketamine)	Yes (Text) No (Text) Unsure Not Reported
Physiological Monitoring During Stroke *If yes, indicate which parameters were monitored*	Yes (Text) No Unsure Not Reported
Was the ischemic stroke injury confirmed via laser Doppler or perfusion imaging?	Yes No Not Reported Unclear
Was there use of a battery of sensory-motor recovery tests? These tests include: 1. Walking Tasks (e.g. Beam, Grid Walking or Ladder Tests) 2. Forelimb Asymmetry Tests (e.g. cylinder tests) 3. Skilled Reaching Tests (e.g. Staircase or Single Pellet Reaching Task) 4. Adhesive Removal Test 5. Neurological Severity Scores (mNSS) 6. Rotarod	Yes: Multiple tests were used No: Only one test was used No: No sensory motor recovery tests were used Unclear
Was the size of infarct proportional to that seen in a human stroke patient?	Yes: Infarct size within reasonable limits (<40%) No: Infarct size was too large (>40%) Unclear: Infarct size was not reported Other (Text)
Did the duration of occlusion create a clinically relevant infarct size?	Yes: Duration of stroke was <90 min No: Duration of stroke was >/= 90 min No: Stroke model was permanent Unclear (Text)

### Exploring the evidence and synthesizing outcome data using network meta-analysis

We will begin by exploring the pattern of treatment comparisons represented by the included set of studies using network diagrams (or using a tabular approach if necessary, should the number and pattern of comparisons be too broad to be summarized graphically). Effect estimates from all included studies will be summarized. We will summarize traits of included studies focusing on clinical (e.g. age, sex, species, stroke model, reperfusion vs. permanent model, comorbidities, severity of infarct pre-treatment, infarct location) ^39^ and methodological (e.g. risk of bias, timing of outcome assessment) features ^27^, and review these with our clinical and preclinical experts to establish the degree of homogeneity within the included studies. For NMA, given the possibility that a large proportion of the studied interventions may have been evaluated in only a single study (and many could potentially yield very large effect sizes, which may not have been substantiated by more animals in more studies), we will exclude these interventions from NMAs performed; each of these treatments removed from NMA will neither benefit from “borrowing strength” through NMA, nor end up with a summary estimate and confidence interval different from what was reported in a single study. The reported findings for the outcomes of interest from studies removed from the NMA according to these criteria will be summarized separately in descriptive tables to ensure all relevant data are summarized. This approach will also restrict the network to a more practical size and reduce the risk of computational challenges.

Where there is homogeneity of important effect modifiers, we will perform NMAs to compare interventions ^9, 10, 40^, following procedures to assess the validity of the assumptions of homogeneity, similarity, and consistency ^41^. Based upon the extracted study characteristics, we will work with our clinical and preclinical experts to establish any additional novel aspects of preclinical studies that may be important to consider in relation to judgements regarding study homogeneity beyond those anticipated in preparing this protocol. We have anticipated different species of animals (rats, mice, gerbils, dogs, sheep, non-human primates) across studies. We also anticipate that multiple reporting formats will have been used to assess both infarct volume (e.g. mm ^3^, % of hemisphere or total brain, etc.) and neurobehavioral changes (e.g. seconds, % of baseline). 

For meta-analysis of preclinical studies, the normalized mean difference (NMD) scale is useful in serving the purpose of synthesizing the complexity of data aforementioned ^42^. Prior to performing NMAs, we will perform traditional pairwise meta-analyses on the NMD scale for each comparison in the treatment networks where two or more studies are available to explore heterogeneity based on the *I ^2^* statistic ^29^. To perform network meta-analyses on the NMD scale, we will use an established model from the National Institute in Health and Care Excellence’s TSD series ^43^, adapting its identity link to the log link in order to conduct the NMA on a log ratio of means (logRoM) scale. The log ratio of means of the k ^th^ treatment and the “stroke only” control, *d _k_* = logRoM *_C,T _k__*, can be estimated after model fitting, and the corresponding NMD estimate is:

                                                                                             1 – exp{logRoM *_C,T _k__*} = 1 – exp ( *d _k_*)

The NMD of the k ^th^ treatment in comparison with alteplase (k=1) is 1 – exp ( *d _k_* – *d*_1_).

Both fixed- and random-effect Bayesian NMAs will be performed using a common heterogeneity parameter according to established methods ^10, 40, 43^. Model fit will be assessed by comparing the model’s posterior total residual deviance with the number of unconstrained data points ^43^. Selection between models will be based on deviance information criteria (DIC), with a difference of five points suggesting an important difference ^43^. All pairwise comparisons between interventions will be expressed with both summary point estimates and corresponding 95% credible intervals. Vague prior distributions will be assigned for all measures of treatment effect, as well as for the between-study variance parameter in random effects analyses. NMAs will be performed using OpenBUGS software version 3.2.3 ^44^ and the R Package R2OpenBUGS ^45^. Model convergence will be assessed using established methods including assessment of Rhat (the potential scale reduction factor) and the Gelman-Rubin convergence diagnostic to see if they are near 1 ^9^. Surface under the cumulative ranking (SUCRA) values, and the mean rank of each intervention (with 2.5% and 97.5% quantiles) will also be estimated for each intervention ^46^. Forest plots of treatment comparisons versus “stroke with no treatment” control as well as versus stroke + alteplase will be prepared for each outcome. Given the anticipated high number of interventions assessed in only a single study, a tabular approach to summarizing findings will be employed for them. We will also undertake forest plots of effects wherein interventions are ordered according to mean rank estimated from NMA.

### Addressing heterogeneity and inconsistency

To check the validity of the consistency assumption (i.e., transitivity of the effect size through common comparators), a consistency model as well as an unrelated mean effects model will be fit to the data ^47^. We will compare their respective DIC values to check model fitting and their posterior mean deviance contribution per study to check the consistency assumption. We will also assess the magnitude of the estimated between-study SD measure from both models, as a reduction in this parameter in the inconsistency model also provides evidence of inconsistency.

The likelihood of important clinical and methodological heterogeneity between studies is anticipated by the research team to be high and may include several nuances which are unique to the pre-clinical setting. First, several vital aspects of preclinical studies from our risk of bias assessments (described earlier) may be important adjustment factors that could have an important impact on the findings from NMAs, including randomization and blinding ^24, 48^. In this work, we will use subgroup analyses or covariate-adjusted analyses to address and explore the impact that covariates have on findings and to establish the robustness of findings from primary syntheses ^49, 50^. We will assess the possibility to adjust for the following group level factors: animal species (e.g. mouse) and strain (e.g. C57Bl6 strain of mice), model of stroke, average animal age, percentage of female subjects, average time since stroke induction, combination therapies, cerebral blood flow, temperature, infarct location and severity, use of randomization and blinding of experimenters and outcome assessments. Alternatively, when combining data from different species, we could model animal species as an extra level in the hierarchical model for treatment effect, allowing for heterogeneity across species and assuming that treatment effects are similar across species around an overall mean effect. For the network structure, primary analyses will be performed at the treatment level. As dose may have an important effect on intervention benefits, we will also explore the range of doses associated with each intervention across studies to consider additional analyses. However, as dose response characteristics of different agents may also vary between animal species and an *a priori* source of information to establish appropriate dose categories is not available, any analyses pursued in relation to dose will be appropriately indicated as post-hoc. Findings from all analyses will be reported. Given the anticipated complexity of this novel application of NMA, we anticipate separate publications will be required for the primary and secondary outcomes.

### Dissemination

The results of the study will be submitted for publication to a peer-reviewed journal and presented at relevant national and international conferences and scientific meetings to promote knowledge transfer.

### Amendments

If amendments are required for this protocol, date of each amendment will be provided with a description for rationale for the change in this section.

## Discussion

Current approaches to evaluating the relative therapeutic benefit of preclinical treatments for stroke are limited. Although systematic reviews have been conducted comparing more than a thousand candidates, many have never been systematically assessed, nor have they been assessed relative to one another, or more importantly, to the best available clinical treatment (alteplase). Use of NMA to synthesize data on all relevant available therapies may help address this knowledge gap. Thinking more broadly, the proposed review, with the application and evaluation of NMA to preclinical therapeutics, will inform translational scientists’ knowledge of which preclinical stroke therapeutics have the most promise for either further preclinical research or translation to clinical trial.

In addition to addressing an important question for clinical research, we anticipate this study will inform empirical explorations of anticipated challenges of evidence synthesis that are unique to the pre-clinical setting. First, a debate among preclinical and clinical scientists is likely to exist regarding both the appropriateness and approach to synthesizing outcome data from different species as well as different models of stroke. Second, there exists an especially important need to consider a broad range of adjustments to account for between-study heterogeneity related to animal characteristics or other features; lack of availability of these key data may be sub-optimal. Our study will provide an empirical evaluation of the degree of missingness of features, such as those significant to experimental design (e.g. randomization). This will provide an indication of the changes to the available evidence when exploring adjustments of comparisons. More specifically, if the lack of reporting proves to be severe, this will provide further high-level evidence that educational efforts are needed to improve the completeness of reporting of preclinical research ^51^.

Other challenges potentially requiring consideration will include identifying optimal strategies for presenting findings (including those with many comparators rendering analysis unfeasible), analysis of studies with small sample sizes, and strategies to select the most promising therapy to translate clinically. We anticipate that this systematic review will provide insight into these and other methodologic challenges and thereby serve as an exemplar for future NMA of preclinical data to build upon.

Findings from this review will be shared with several key knowledge users including (i) the Stroke Treatment Academic Industry Roundtable ^52^ for development of future guidelines; (ii) the Heart & Stroke Foundation and the Canadian Partnership for Stroke Recovery to inform future potential trials ^51^; (iii) the Cochrane Stroke Group to inform a future clinical systematic review and NMA; and (iv) stroke survivors, via sharing of findings with our knowledge users.

## Data availability

No data are associated with this article.

## Supplementary materials

**Supplementary File 1. PRISMA-Protocols checklist.**

**Supplementary File 2. Representative search strategy.**
