# Supplementary material for: Identifying stroke therapeutics from preclinical models: A protocol for a novel application of network meta-analysis
Source: F1000Res. 2019 Jan 3;8:11. [Version 1] doi: 10.12688/f1000research.15869.1 (PMC6426098; doi:10.12688/f1000research.15869.1)
Supplement: Peer review history

# Peer review: Reviewer response for version 1

**DOI:** 10.5256/f1000research.17325.r44938

**Published:** 2019-03-19

**Authors:** Hanna M. Vesterinen

**Affiliations:** 1 Scientific Consultant, University of California, San Francisco, California, CA, USA

This is a fascinating research question and the methods set out in this protocol seem appropriate. I am satisfied that the authors have set out the protocol in accordance with the PRISMA-P checklist.

 I have outlined a few points below:

1. I think the decision to reject studies which deliver therapies outside a 6 hour window warrants some additional background information. This may well be an appropriate decision; however, I don't have expertise in stroke and so it leads me to question if there is the *possibility* that the combination therapies could lead to greater efficacy/less harm outside this time period. 
2. In table 2, will the authors state what "other" is for species and type of model? This seems like useful information.
3. Table 2 part b, should it state "N Initially Reported"?
4. Is "potential bias due to sample size calculation" actually related to imprecision rather than risk of bias? 
5. Dichotomous cut offs might lose valuable information e.g. infarct <40% is within reasonable limits vs >40% is not. Is 39% vs 41% really that different?

I have read this submission. I believe that I have an appropriate level of expertise to confirm that it is of an acceptable scientific standard.

# Peer review: Reviewer response for version 1

**DOI:** 10.5256/f1000research.17325.r42520

**Published:** 2019-01-28

**Authors:** Peter-Paul Zwetsloot

**Affiliations:** 1 Department of Cardiology, Experimental Cardiology laboratory, University Medical Center Utrecht, Utrecht, The Netherlands

Lalu et al. provide us with their research protocol for a network meta-analysis of novel stroke therapeutics in preclinical models. The protocol is comprehensive and complete, including all necessary items like search strategy, screening and extensive data analysis. The search seems complete, using an animal filter and searching the Embase library, including Medline through Embase. 

 There are some minor concerns and suggestions for this paper and approach to potentially be more optimal; 

- The paper describes both fixed and random effects meta-analysis. As a network meta-analysis already usually is performed with random effects and your expected variation is considerable, I would skip the fixed effect meta-analysis as a whole. 
- Even when using an NMD, is it appropriate to combine MRI and histology based outcomes, as these are known to not generate equivalent outcomes in preclinical models (MRI > histology, see Milidonis, Stroke 2015)? Will the NMD completely correct for this or is a sensitivity analysis needed (MRI vs histology)? If you don't think a sensitivity analysis is needed, please explain why not.
- Will the NMD also be used for the secondary outcome? This is not completely clear to me now. 
- Consider to not dichotomise certain potential effect modifiers (for example the analysis time < or >30d as mentioned on page 4). Sometimes a continuous variable can give you more information in your analysis (for a potential linear effect for example). You can also choose to do both. 
- Following on the previous comment; network meta-analysis is usually performed through a form of metaregression, making it possible to correct (potentially mutivariably) for a number of potential confounders/effect modifiers in the primary analysis itself. This is already mentioned on page 12 for the 'covariate-adjusted analyses'. 
- Please provide a list upfront of the potential factors you want to correct for (in order of importance/usage) and provide an explanation on the number of factors you want to correct for (potentially based on the number of included studies?). To my knowledge this is different form the stated 'review these with our clinical and preclinical experts to establish the degree of homogeneity' and would add to your future primary analysis. This also means that the studies does not necessarily need to be homogenous for your primary analysis, as the metaregression will appoint a certain effect to these 'covariables' (and will correct for the covariable). 
- Please provide a minimum number of comparisons for a certain intervention/comparison to be included in the network meta-analysis. Will there also be 2 or more, as with the traditional pairwise meta-analysis mentioned? If no minimum can be mentioned upfront, please explain why. 

I have read this submission. I believe that I have an appropriate level of expertise to confirm that it is of an acceptable scientific standard.
